# HADA: A Hybrid Authentication and Dynamic Attribute Access Control Mechanism for the Internet of Things Using Hyperledger Fabric Blockchain

**DOI:** 10.3390/s26082531

**Published:** 2026-04-20

**Authors:** Suhair Alshehri

**Affiliations:** Information Technology Department, Faculty of Computing and Information Technology, King Abdulaziz University, Jeddah 21589, Saudi Arabia; sdalshehri@kau.edu.sa

**Keywords:** attribute access control, authentication, Hyperledger Fabric blockchain, Internet of Things, lightweight algorithm

## Abstract

The proliferation of Internet of Things (IoT) devices has created unprecedented challenges in cybersecurity, as billions of interconnected devices generate, process, and transmit sensitive data across diverse networks. This study addresses critical security vulnerabilities in IoT ecosystems, focusing on the development of a comprehensive security framework that encompasses device authentication, an attribute access control mechanism, and privacy preservation. This work introduces HADA, a proposed hybrid authentication method that combines the validation of unique credentials and trust value. For the authentication of the data owner and user, the following credentials are validated: identity, certificate, reconfigurable physical unclonable function (PUF), and trust. Differential privacy is used to secure the credentials during information exchange. Then, the newly developed dynamic attribute access control method selects the number of attributes and matches the attributes; these two processes are performed using the Bi-Fuzzy logic and graph neural network (GNN) algorithms, respectively. After matching the data, the user is allowed to access them from the cloud server. For data encryption, the lightweight SKINNY algorithm is implemented in Hyperledger Fabric blockchain. The proposed system performs better than existing methods in terms of throughput, latency, and resource utilization.

## 1. Introduction

With the digital revolution, Internet of Things (IoT) technologies have been developed to interconnect millions of devices and facilitate effortless communication. IoT systems have evolved to incorporate machine learning, artificial intelligence, and deep learning methodologies [1,2,3]. The IoT is an environment in which the devices can generate, share, communicate, and consume data with minimal human monitoring. It enables the development of new applications for quality improvement in smart homes, industries, healthcare, and city appliances. The fine-tuning of multiple features allows for the resolution of scalability issues that eventually result in security issues. Alongside the IoT, blockchain technology is a promising solution for security [4,5,6].

The combination of the IoT and blockchain technology assures security for all resource-constrained devices with efficiency and transparency. Blockchain operates in a decentralized manner that assures communication between millions of smart devices. Security is a major concern in the incorporation of IoT applications, particularly regarding cyberattacks. Blockchain is a peer-to-peer network that stores transactions sequentially in hash values; it comprises a system of computers in a network that records the transactions securely as a digital ledger. Highly immutable features in blockchain enable one to confirm the recorded transactions, which cannot be modified or deleted. Blockchain can store millions of transactions and can be accessed at any time. The consensus algorithm, which is responsible for validating the blocks to maintain secrecy, plays a vital role. Data consistency and fault tolerance are the two key features of the consensus algorithm that increase the utilization of blockchain.

A critical aspect of security is mitigating attacks and eliminating illegitimate users in the system [7,8]. Authentication is the best strategy for allowing only legitimate users entry according to the registered information [9], acting as a first line of defense to shield credentials and data. At present, all applications require security due to the sharing of confidential information [10,11,12,13]. Devices in the IoT system have heterogeneous resource specifications, making providing security a complex task. Multiple types of attacks have emerged with the aim of modifying information, obtaining extracted data, compromising devices, and so on. Increases in privacy concerns often require device authentication, which helps to restrict access to legitimate devices.

The digital revolution has resulted in many security solutions, such as two-factor authentication, multi-factor authentication, lightweight cryptography, hashing, the zero-trust system, and the intrusion detection system, facilitating secure registered information and user data. The attribute-based access control (ABAC) mechanism is also used to improve security when allowing users to extract the stored data [14,15,16,17]. Large-scale organizations make data access flexible with an attribute access control mechanism. The ABAC mechanism comprises a set of attributes that can be of any of the following types: user attributes, object attributes, action attributes, and environmental attributes. Matching occurs according to the list of attributes, and a decision to allow or deny access is made [18,19,20,21].

The increasing popularity of the IoT entails security requirements, especially access control that permits access for authorized devices. Many solutions are available for managing access control with perfect decision-making. In general, the attributes are categorized as subject-based, environment-based, time-based, originality-based, or authority-based. According to the IoT application, an attribute is chosen, and actions are taken. Common challenges associated with IoT applications are as follows:

- Implementing hashing and cryptographic algorithms with higher computational requirements unsuitable for resource-constrained IoT devices.
- Defining policies based on attributes for devices and users in a scalable manner is challenging.

In this study, an authentication and access control mechanism is developed to provide security for data owners and users. The IoT environment can collect data, manage it, and store it. Then, the stored data can be accessed by each data user, which aids analysis and decision-making. The method uses Hyperledger Fabric (HLF) blockchain.

The key objectives of this research are as follows:

- To implement a dynamic attribute selection framework that adapts to changing data patterns and user requirements in real time.
- To optimize system performance through parallel processing techniques in deep learning models, enabling efficient handling of large-scale attribute selection and access control decisions across distributed environments.
- To develop a robust authentication framework that combines traditional user security credentials with dynamic trust evaluation to enhance the security and reliability of access control systems in distributed environments.
- To design and implement a lightweight hashing mechanism within the HLF blockchain framework that optimizes resource utilization while maintaining security guarantees, with the aim of improving performance in resource-constrained environments.

### 1.1. Contributions of This Paper

The key contributions of this paper are as follows:

- To prevent the participation of illegitimate data owners and users, a multi-level authentication framework is developed using identity verification, digital certificates, and Re-Physical Unclonable Function (PUF)–based device authentication, followed by trust validation. The authenticated data owner employs the lightweight SKINNY encryption algorithm to securely encrypt the data prior to cloud or network upload.
- To enhance the integrity and confidentiality of user and owner credentials, the lightweight SPONGENT hashing algorithm is integrated within the HLF blockchain environment, enabling secure and tamper-resistant storage of credential hashes.
- To support adaptive and context-aware attribute selection, a dynamic attribute-based access control (ABAC) mechanism is designed using a Bi-Fuzzy Q-Learning approach. As multiple users with varying requirements interact within the system, the model selects an optimal set of access attributes for each time period, ensuring flexible, fine-grained, and intelligent access management.

Compared with existing blockchain-based IoT security methods, the novelty of the proposed HADA framework lies in combining multi-level authentication using identity, certificate, Re-PUF, and trust validation with dynamic attribute-based access control in a single permissioned Hyperledger Fabric environment. In particular, the proposed framework is intended to address the limitations identified in previous work, where authentication often relies on either static credentials or trust evaluation alone, and access control commonly validates a fixed set of attributes for each request. Thus, HADA is integrates both static and dynamic security factors within one framework while supporting dynamic attribute validation.

### 1.2. Organization of This Paper

This remainder of this paper is organized as follows: Section 2 discusses state-of-the-art works and their limitations and drawbacks, Section 3 reports problems identified in previous research, Section 4 explains the proposed algorithm that solves the predicted problems, Section 5 provides experimental results with comparisons to prove the efficiency of the proposed system, and Section 6 provides conclusions and with future research directions.

## 2. Related Works

In this section, existing research methodologies are examined and their drawbacks are discussed, considering authentication and attribute access control mechanisms using blockchain technology.

A previous study presented autonomous truck platooning with a quantum-safe blockchain-empowered authentication mechanism [22]. This system was composed of permissioned blockchain, certificate authority, trucking firms, and autonomous trucks. The authors presented a short-distance communication method in which keys were generated for a group of trucks and verified using the unique signature of each user. As multiple trucks were involved, signature aggregation was validated in blockchain. A signature was generated for a group of vehicles but was unable to indicate if any specific illegitimate vehicle was present in the group. Using lightweight authentication mechanisms and optimized storage, the data were uploaded using fully homomorphic encryption [23]. Initially, a cluster head was selected based on battery power. Each IoT device sent a request to a service provider, which validated the smart contract and then accepted or declined access control for that device.

The authors of Ref. [24] developed a two-factor authentication method that incorporated blockchain technology to enhance traditional authentication. Two credentials, username and password, were considered for authentication, but these are common credentials that can be hacked by attackers. An HLF blockchain-based authentication scheme was presented as a multiserver architecture [25]. According to this scheme, the server and user register, after which authentication is performed with a smart card, identity, password, and biometrics. Although multiple factors were involved in authentication, the smart card was a poor choice because it can be stolen and because anyone who holds the smart card can be authenticated.

Other authors presented an HLF blockchain framework and attribute-based access control [26]. Their system architecture was composed of users, blockchain, a smart gateway, and devices. Blockchain was the intermediate entity that validated the smart contracts and processed the attribute access control mechanism. For security, unique certificates were generated by a certificate authority and validated. The Message Queuing Telemetry Transport (MQTT) protocol was used because it can be employed between IoT devices. The authors of Ref. [27] presented a blockchain technology with a machine learning algorithm that supports healthcare application. Healthcare data were collected from smart phones, wearable IoT devices, and a diagnostic center. The patient details were registered individually with specific credentials, after which insurance claims could be made. Insurance policy verification was performed, and insurance fraud was detected using a machine learning algorithm. The Support Vector Machine (SVM) was used, and classification was performed. However, use of the SVM algorithm extended the training time, making it unsuitable for a scalable system environment.

An improved blockchain and elliptic-curve-based multi-authority attribute access control scheme was developed to reduce tampering attacks and data leakage [28]. This approach also minimizes single-point failure via the incorporation of distributed storage. This system architecture was designed with a certificate authority, interplanetary file system (IPFS), attribute authorities, data owner, data visitor, and HLF blockchain. In this work, the attribute authority generates a key pair for each attribute, which is validated in the blockchain. The SHA-1 and AES algorithms were used for hashing and cryptography and require more computation than lightweight algorithms.

A blockchain-based system was developed for an e-health system application that provides privacy-preserving authentication and an access control scheme [29]. This system was designed to operate in six consecutive layers: the user layer, authentication layer, access control layer, blockchain layer, data storage layer, and healthcare application layer. Access control was enabled using smart contracts with pre-defined policies for user roles with different access levels: doctors, laboratories, devices, and third-party users. This approach uses blockchain and the SHA-256 hashing algorithm, which requires heavy computation and is therefore unsuitable for scalable systems.

In Ref. [30], a zero-trust access control mechanism for the IoT in a 6G environment was presented. This approach also employs smart contracts for access control and builds a trust model using a consensus algorithm. The key entities involved in this system model are an interplanetary file system, policy generation contract, policy decision contract, and trust mechanism contract. According to this work, the access control policies can be altered dynamically, and, identity was the single credential used for authentication. When using a single credential for authentication, the risk of attack is higher. The HLF access control scheme was proposed in this work.

An access control system was presented for Industrial Internet of Things (IIoT) [31]. For this attribute-based access control method, three smart contracts were developed: device, policy, and access contracts. The policy contract was enabled to define policies for the administrator and data consumer. The device contract was used for storing the URL and device identity. Finally, the access contract was implemented between the subject and object and was responsible for decision-making regarding access control. According to this contract, access was provided for users. Efficient file sharing in a decentralized environment in HLF blockchain using attribute-based encryption was proposed [32]. Based on this encryption, legitimate data owners were only allowed into the system for processing. This approach had four main steps: setup, encrypt, key generation, and decrypt.

Other recent studies [33,34,35] have also investigated blockchain-assisted IoT access control using message queuing support, dynamic, secure medical data access frameworks, and blockchain design trade-offs in IoT-oriented environments, further confirming the importance of scalable and secure access control in distributed IoT systems.

The state-of-the-art methods reviewed above share limitations and drawbacks in IoT authentication and attribute access control methods, for which solutions have been proposed.

## 3. Problem Statement

A trust-based authorization access control (TAAC) mechanism that evaluates trust values using a dynamic trust calculation model has been proposed [36]. The key components involved in this system model are registered authority, a hospital, doctors, and a dynamic trust calculation model. Trust is estimated using the Eva algorithm, which considers direct trust, indirect trust, historical trust, and biased trust. A signature is generated from the trust value and converted into hash values for security. Upon verification of the hash value, the user is allowed access. This approach requires considerable resources (energy) for mining blocks from the blockchain, and the Eva algorithm used for trust computation is vulnerable to attacks from certain malicious nodes, such as collusion attacks and Sybil attacks.

A novel Trust-based Access Control Mechanism using permission hyperledger blockchain technology (TABI) was proposed [37]. A trust calculation contract was implemented on edge devices to ensure security. The main aim of this work was to reduce the involvement of malicious IoT users and devices. Nodes with lower trust values were identified as malicious, whereas higher trust values indicated legitimate nodes. According to the smart contract, the trust values were validated and updated. The problems stated are as follows:

- Trust value detection is based on false positive and false negative values. However, the node’s behavior and node communication are important for identifying whether a particular node is legitimate or malicious.
- In general, smart contracts are static and cannot be modified, so the trust calculation contracts developed cannot be the same for the devices at all times as the trust value of a device will be updated dynamically according to its behavior.

A secure and dynamic access control scheme (SDACS) was proposed in [38]. The authors of this study proposed a certificateless authentication protocol comprising a trusted central authority, data owner, data user, blockchain, and Interplanetary File System (IPFS). This approach involves five processes: registration, data encryption, blockchain initialization, access control, and data decryption. In this research work, the blockchain technology uses SHA256 hashing, which employs larger hashes to increase storage and consumes more processing power, making it un suitable for resource-constrained devices as IoT devices. User identity is the only credential considered during registration and is used for authenticating the user. In blockchain, conventional hashing is employed using the SHA-256 algorithm, and larger hashes are retrieved, which increases the computational burden and, consequently, the complexity. The problems stated in previous research works are as follows:

- The traditional algorithm SHA-256, used in the blockchain, requires multiple computations that slow the system when used continuously. It operates slower than lightweight hashing algorithms that increase latency.
- In previous research works on authentication, either credentials are validated or the trust value is estimated for validation. However, when the credentials are stored once the user is registered while the trust value is estimated dynamically according to activity, both are significant in authentication, an approach that has been unsuccessful in previous research.
- The attribute access control mechanisms consider a set of attributes which are always validated every time the user requests access; an increase in the number of attributes will increase time to validation.

The key limitations of previous research works reported in this section are challenging to overcome. The aim of the method proposed in this study is to solve these issues via a HADA system combining hybrid authentication and dynamic attribute access control.

## 4. Proposed HADA Control Mechanism

### 4.1. HADA System Model

**Figure 1:**
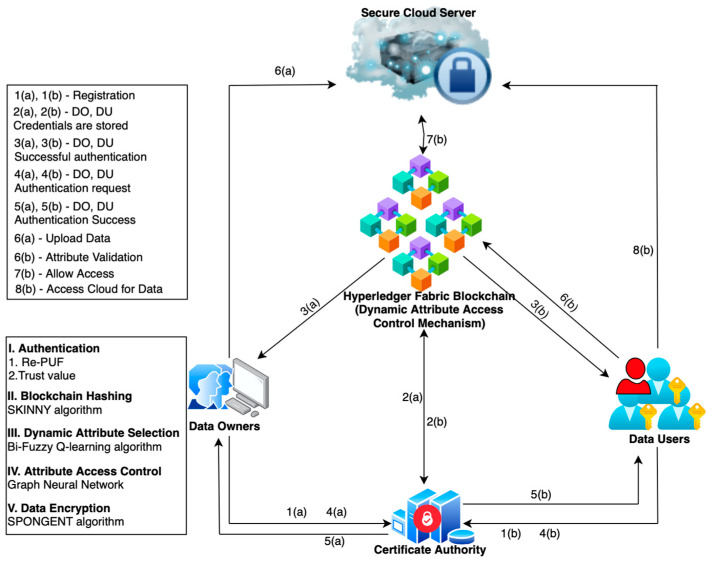
HADA System Model.

The proposed HADA model, illustrated in Figure 1, is composed of the following entities: the data owner (DO), data user (DU), certificate authority (CA), HLF blockchain, and cloud. The data owner has the right to upload and download the data, whereas the data user has the right to view and download the data. Both the DO and DU must register first. Authentication is required to access the cloud. The CA generates certificates and then validates them during authentication. The security credentials are maintained in HLF blockchain, which is an open-source platform that has a decentralized ledger. Let the number of DOs and DUs be denoted as $D_{N}=D_{1},D_{2},\ldots \ldots ,D_{N}$ and $U_{M}=u_{1},u_{2},\ldots \ldots ,u_{m}$, where N and m are the total number of DOs and DUs.

A DO is an entity with the rights to upload any IoT data to the cloud, and the DU can access the data once successfully authenticated. The CA is responsible for generating unique certificate for each DO and DU that cannot be altered easily. It validates the entire registration process for both the DO and DU. All registered security credentials are updated to the HLF blockchain, which stores hashes in blocks that are used during authentication. The attribute access control mechanism matches the attributes for each request and allows access.

In a real workflow, the data owner and user first register with the certificate authority by submitting identity-related credentials. The certificate authority generates and validates the certificate information, while the corresponding hashed authentication credentials are maintained in the Hyperledger Fabric blockchain. During authentication, the certificate authority verifies the submitted credentials and cross-checks the blockchain-stored hashes. After successful authentication, the data owner is allowed to encrypt and upload data into the cloud, and the data user is allowed to request access. Then, the dynamic attribute-based access control mechanism validates the selected access attributes and makes the final decision to allow or deny access.

In the proposed framework, the certificate authority is responsible for credential issuance and registration support, while the Hyperledger Fabric blockchain maintains the corresponding hashed authentication records through distributed peer validation. In this way, identity establishment remains CA-assisted, whereas integrity protection and record validation benefit from the decentralized properties of the permissioned ledger.

Each component in the proposed HADA framework has a specific role. Differential privacy is used to protect sensitive metadata during credential exchange. Re-PUF-based challenge–response validation strengthens device-level authentication. The trust value is included because the behavioral status of the node may change over time while registered credentials remain valid. The SKINNY algorithm is used to encrypt uploaded data with a lower computational burden, and SPONGENT is used for the lightweight hashing of credentials in the blockchain layer. Finally, the dynamic attribute-based access control mechanism is used to support context-dependent access validation according to the request conditions.

### 4.2. DO and DU Registration and Authentication

The data owner and user register with the identity, certificate, and reconfigurable Physical Unclonable Function (Re-PUF). These credentials are unique for each owner and user that participate in the system. The HLF blockchain stores the credentials in hashes and validates them during authentication. After authenticating the credentials, trust values are estimated, and it is verified. Upon successful authentication, the DO is allowed to upload data, which are encrypted using the lightweight SKINNY algorithm.

In this work, Re-PUF is considered a device-authentication primitive whose challenge–response set can be refreshed or updated during the device’s life cycle. Therefore, the challenge–response entries shown in Table 1 are only illustrative examples of registered pairs and do not imply that the same static pair is reused permanently. The reconfigurable aspect refers to updating the active challenge–response information during subsequent controlled registration or credential update operations.

#### 4.2.1. Data Owner Registration

Step 1: Consider $D_{1}$ for registration; it sends a request to the CA with an identity and timestamp. The request message is $R_{1}\to \left\{{ID}_{1},t_{0}\right\},$ where $R_{1}$ is the request, ${ID}_{1}$ is the identity of the DO, and $t_{0}$ is the initial timestamp.

Step 2: The CA checks the timestamp $t_{0}<∆T$; if valid, the identity of the DO will be stored, and the CA will generate certificate $F_{1}$. In this way, the certificate will be generated for all $D_{N}$. The certificate is secured using differential privacy and gives the response $R_{1}\to \left\{{ID}_{1},\eta \left(F_{1}\right),t_{1}\right\}$, where $F_{1}$ is the generated certificate for $D_{1}$, and η represents the noise added as metadata. Differential privacy is the concept of applying noise to data as follows:

Step 2.1: Let $F_{1}$ the sensitive data to which Laplace mechanism-based noise is to be added.

Step 2.2: The Laplace noise is as follows:

(1) η~Laplace(0,∆f/ϵ)

where ϵ is the privacy budget that can be set to 0.1, and ∆f is the sensitivity of the data. The digital certificates are generated in the form of binary bytes. According to the proposed approach, differential privacy operates based on different layers, and the noise is added to the metadata and not the certificate to ensure protection for the certificate. The metadata is associated with the user’s identity and certificate number.

Laplace Mechanism is in Algorithm 1:
**Algorithm 1.** Laplace Mechanismadd_laplace_noise(true_value, sensitivity, epsilon): # Scale parameter is sensitivity/epsilon scale = sensitivity/epsilon # Generate noise from Laplace distribution noise = np.random.laplace(0, scale) # Return noisy value return true_value + noise

Step 3: $D_{1}$ checks ${(t}_{1}-t_{0})<∆T$; if the timestamp is true, then it sends a set of challenge–response pairs as a request to update it. $R_{2}\to \left\{\left(C_{1},R_{1}\right),\left(C_{2},R_{2}\right),\ldots ,\left(C_{k},R_{k}\right)\right\},t_{2}$, the challenge–response pairs, are as shown in Table 1.

**Figure 2:**
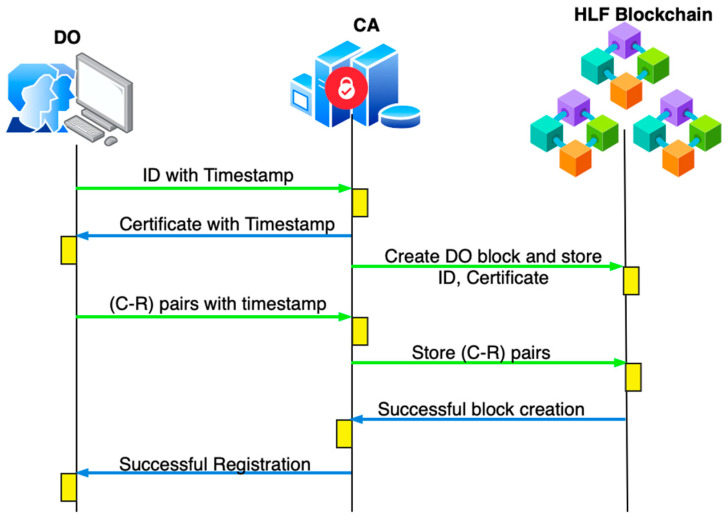
Workflow of DO registration.

Step 4: The CA checks ${(t}_{2}-t_{1})<∆T$; if the timestamp exists, it updates the credentials in the HLF blockchain. With these credentials, the registration process is completed. A notification of successful registration is provided as the response $R_{2}\to \{Reg\;Success\}$. The data owner is now ready to upload data after completing authentication. The complete registration workflow is illustrated in Figure 2.

#### 4.2.2. Data Owner Authentication

Authentication is handled with the registered credentials. The steps involved in authentication between CA and $D_{1}$ are provided below.

Step 1: Let $D_{1}$ be the owner sending a request to CA, $R_{1}\to \left\{\left({ID}_{1}\right),t_{0}\right\},$ for authentication to upload data to the cloud.

Step 2: The CA receives the request and first validates the timestamp as $t_{0}<∆T$ and checks the identity. Here, ∆*T* is the threshold timestamp. If the identity exists, the certificate is required for the next step of authentication. The response is returned to the data owner as $R_{1}\to \{{ID}_{1},\left(F_{y}\right),t_{1}\}$.

Step 3: Upon receiving the response, $D_{1}$ extracts the timestamp and checks $(t_{1}-t_{0})<∆T$; if true, it sends the certificate with noise added as metadata to the CA in the form $R_{2}\to \{({ID}_{1}),{\eta \left(F_{1}\right),t}_{2}\}$.

Step 4: The CA receives $R_{2}$ and validates the timestamp; if $\left(t_{2}-t_{1}\right)<∆,$ it checks the certificate and, if valid for the corresponding $D_{1}$, creates $R_{2}\to \{{ID}_{1},\left(C_{y},R_{y}\right),t_{3}\}$.

Step 5: If the timestamp $(t_{2}-t_{1})<∆T$ is valid, $D_{1}$ selects any of the pairs and sends $R_{3}\to \{\left(C_{1},R_{1}\right),t_{3}\}$ to the CA.

Step 6: The CA forwards $R_{3}$ to the HLF blockchain to validate the hashed $\left(C_{1},R_{1}\right)$. CA checks $\left(t_{3}-t_{2}\right)<∆;$ if it is true, then the HLF blockchain validates $\left(C_{1},R_{1}\right)$ based on the status action in Table 2. After validation of this credential, the data can be uploaded, and the message $R_{3}\to (Auth\;Success,\;t_{4})$ is sent to $D_{1}$ with the timestamp.

Step 7: $D_{1}$ checks $\left(t_{4}-t_{3}\right)<∆T$, encrypts the data using the SKINNY algorithm, and uploads it to the cloud.

**Figure 3:**
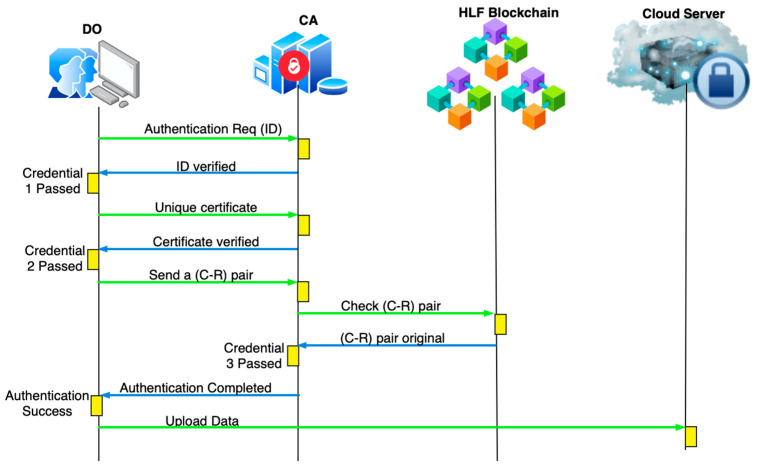
Workflow of DO authentication.

The authentication workflow is depicted in Figure 3. The SKINNY algorithm starts with the initialization of the state and plain text as (S,PT). The plain text is the data that must be uploaded to the cloud. The algorithm takes plain text and the key for encryption. Initially, the (S,PT) is XORed to the initial round key $K_{0}$; then, the TweaKey (TK) schedule is applied to multiply the round keys, also generating subkeys using tweakey input. The tweakey update function is defined as follows:

(2) $$K_{i+1}=f(K_{i},T_{i})$$

where $T_{i}$ is the round-dependent tweak component that is required tweakey schedule during $i^{th}$ round. For encryption, tweakable block cipher rounds are executed based on a substitution–permutation network in the following four steps:

(i) Add Round Key: In this step, the current state is combined with the round key using the bitwise exclusive-OR operator. Let the $K_{i}$ be the round key for this current round, which is
  (3) $$S^{\prime }=S⊕K_{i}$$
  where S is the current state matrix, $K_{i}$ is derived from the key-scheduling algorithm, and ⊕ is the bitwise XOR operator. This step is significant independent of the secret key, and a single change in the key is reflected in the cipher text.
(ii) Sub-Cells: In this step, a 4-bit substitution-box (S-box) is considered for each state which is non-linear:
  (4) $$S^{\prime }=SBox(S)$$
  It maps each 4-bit input to the 4-bit output, facilitating resistance to differential and linear cryptanalysis. Let $s_{j}^{\prime }$ be the individual 4-bit cell, which is mathematically represented as follows:
  (5) $$s_{j}^{\prime }=SBox(s_{j})$$
(iii) Shift Row: The diffusion is increased based on the performance of rotation on the row of state matrix.
  (6) $$S^{\prime }=SR(S)$$
  The state matrix is given as
  (7) $$S=\left[\begin{array}{cccc} s_{0,0} & s_{0,1} & s_{0,2} & s_{0,3}\\ s_{1,0} & s_{1,1} & s_{1,2} & s_{1,3}\\ s_{2,0} & s_{2,1} & s_{2,2} & s_{2,3}\\ s_{3,0} & s_{3,1} & s_{3,2} & s_{3,3} \end{array}\right]$$
  The rows are shifted from one position to another; as a result, this operation distributes each byte across more than one column and enriches inter-column dependency.
(iv) Mix Columns: The mix of columns on any single bit is reflected in the state of input. Maximum Distance Separable (MDS) matrix transformation is executed as follows:
  (8) $$S^{\prime }=MDS(S)$$
  Finally, r rounds are completed, and the output is cipher text, CT:
  (9) $$CT=S_{r}$$

The generated MDS ensures robust differential privacy, and the single-bit modification in the input completely alters all the bits of the output. The primary reason for choosing SKINNY over AES for our framework is its suitability for resource-constrained IoT devices. While AES is a highly secure and widely standardized block cipher, its implementation typically requires greater computational resources, memory footprint, and power consumption compared to lightweight ciphers.

#### 4.2.3. Data User Registration

Step 1: Let $u_{1}$ be the data user requesting registration, which sends $R_{1}\to \{u{id}_{1},t_{0}\}$ to the CA.

Step 2: The CA validates the timestamp $t_{0}<∆T$, stores the identity, and generates a certificate for it. The generated certificate is secured with differential privacy using the Laplace mechanism. Then, the response $R_{1}\to \{{uid}_{1},\left({uF}_{1}\right),t_{1}\}$ is sent to the data user, where ${uF}_{1}$ is the noise added in the form of metadata.

Step 3: $u_{1}$ checks ${(t_{1}-t}_{0})<∆T;$ if the timestamp is true, then it extracts the certificate and request with a challenge–response pair to be stored in the HLF blockchain. The request message $R_{2}\to \{\left(c_{1},r_{1}\right),t_{2}\}$ is sent to the CA.

Step 4: The CA receives $R_{2}$ and checks ${(t_{2}-t}_{1})<∆T$. After confirming the timestamp, it forwards $\left(c_{1},r_{1}\right)$ to the HLF blockchain as $\left\{u{id}_{1},{uF}_{1},\left(c_{1},r_{1}\right)\right\},$ for which the HLF blockchain creates a block to store the data user’s details. All challenge–response pairs are stored here so they can be matched during authentication. The CA sends a response to the user confirming that registration was successful: $R_{2}\to \{\left\{Reg\;Success\right\},t_{3}\}$.

**Figure 4:**
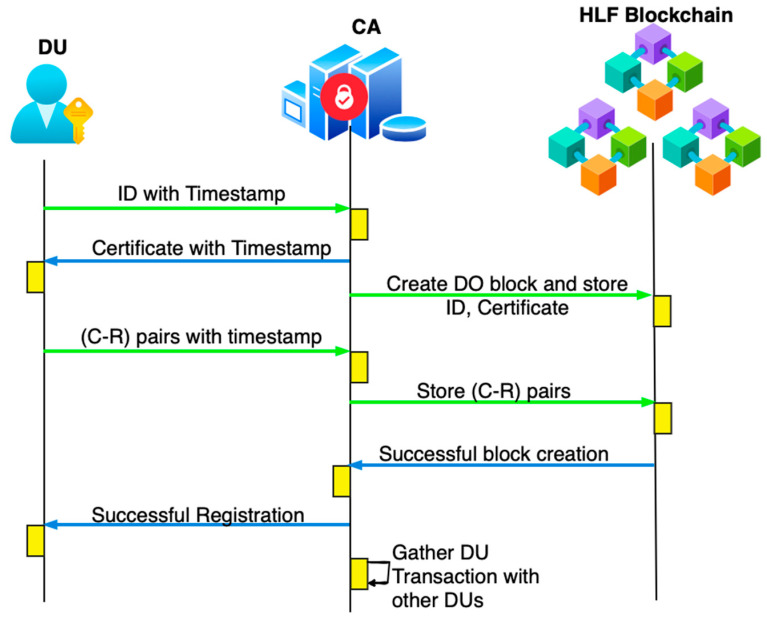
Workflow of DU registration.

Step 5: The data user receives a success message regarding registration and is ready to access the uploaded data from the cloud after successful authentication. Figure 4 shows the DU registration workflow.

#### 4.2.4. Data User Authentication

Step 1: Consider $u_{1}$ to be the data user requesting to access the data by sending the authentication request ${AR}_{1}\to \{\eta (u{id}_{1}),t_{0}\}$ to the CA. Here, AR denotes the authentication request, and the transmission of each credential includes noise for security.

Step 2: The CA receives the request and extracts the timestamp. If $t_{0}<∆T$ is true, it generates a response to validate the certificate to continue the authentication. The authentication response to the data user is of the form ${AR}_{1}\to \{{id}_{1},\left(F_{x}\right),t_{1}\}$.

Step 3: $u_{1}$, upon receiving the response, extracts the timestamp and checks $\left(t_{1}-t_{0}\right)<∆T;$ if true, it sends the certificate: ${AR}_{2}\to \{{id}_{1},\eta \left(F_{1}\right),t_{2}\}$.

Step 4: The CA checks if $\left(t_{2}-t_{1}\right)<∆T;$ then, it validates the certificate corresponding to the $u_{1}$. Upon verifying that the credential is original, it proceeds with the next response, ${AR}_{2}\to \left\{{id}_{1},\left(C_{x},R_{x}\right),t_{3}\right\},$ to $u_{1}$.

Step 5: $u_{1}$ checks $\left(t_{3}-t_{2}\right)<∆T;$ if the timestamp is true, it selects any one of the pairs and adds noise using differential privacy. It then sends ${AR}_{3}\to \{{id}_{1},\eta \left(C_{1},R_{1}\right),t_{4}\}$ to the CA.

Step 6: The CA forwards ${AR}_{3}$ to the HLF blockchain to validate the $\left(C_{1},R_{1}\right)$. The CA checks $\left(t_{4}-t_{3}\right)<∆T;$ if it is true, then the HLF blockchain validates $\left(C_{1},R_{1}\right)$ based on the status action in Table 2. After validating this credential, the HLF blockchain sends the status to the CA in the form $AR_{2}\to (Auth\;Success)$. It responds with ${AR}_{3}\to \{{id}_{1},\left({⋎}_{x}\right),t_{5}\}$ for the trust value verification.

Step 7: The $u_{1}$ checks $\left(t_{5}-t_{4}\right)<∆T$ and determines the trust value as follows: in response to the CA, the trust value ⋎ is computed for $u_{1}$ based on the formula

(10) $$⋎={DT}_{x}+{IT}_{x}$$

where ${DT}_{x}$ and ${IT}_{x}$ represents direct trust and indirect trust of the IoT device. Here, direct trust reflects behavior observed from the node’s own interactions, while indirect trust reflects trust-related information obtained from other entities in the network environment. These values are updated according to node behavior and are checked during the authentication process. The simplified formulation used in this work was chosen to maintain lightweight operation in the simulated IoT setting. If ⋎ is higher than the threshold value, then the data user is legitimate and is allowed to access data from the cloud. It sends ${AR}_{4}\to \{{id}_{1},{⋎}_{1},t_{6}\}$ to the CA.

**Figure 5:**
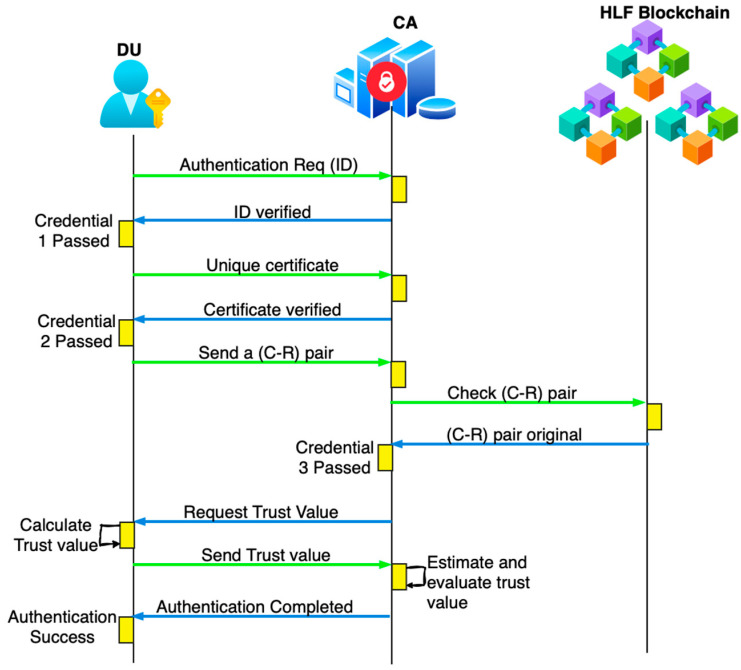
Workflow of DU authentication.

Step 8: The CA checks $\left(t_{6}-t_{5}\right)<∆T$; if true, then the ${⋎}_{1}$ is cross-verified and predicted to be correct, and it sends the successful authentication response ${AR}_{4}\to \{{id}_{1},Auth\;Success\}$. It then requests access to the cloud for specific data. The workflow of DU authentication is shown Figure 5. For authentication, Re-PUF is introduced; it is superior to PUF as it can change and update the challenge–response pair, which cannot be compromised, and it consumes less power. Trust estimation is then included to represent the individual behavior of the user. In the proposed approach, we validate the credentials and trust to ensure that only legitimate users have access.

### 4.3. Dynamic Attribute Selection and Attribute-Based Access Control

In this work, a novel dynamic attribute selection and attribute-based access control scheme, using Bi-Fuzzy Q-learning and a Graph Neural Network (GNN), respectively, is proposed. The motivation behind this dynamic selection was to avoid validating the same full set of attributes for every request, which would increase time and processing overhead. Instead, all the attributes or exactly half of the attributes are selected for validation before allowing access. This design demonstrates dynamic access-control behavior with minimal decision overhead in the simulated IoT environment. A more fine-grained multi-level or fully flexible attribute subset selection strategy is left for future work. For the selection of attributes, the parameters taken in account are location, time, role in the system, and frequency of usage. Then, according to the selected number of attributes, the GNN is applied to validate the attributes for each user request and allow or deny access.

Dynamic attribute selection is the selection of the number of attributes that are to be matched for permitting users. After selection, the set of users arrive at particular time period. Therefore, the attributes set are chosen for each time period. In the proposed approach, two fuzzy logic systems work in parallel with a set of inputs as two. Let x,y,p,q represent the user location, login time, user role, and used frequency. The fuzzy logic is combined with Q-learning for setting dynamic rules according to the environment. The steps involved in this algorithm are fuzzification, rule evaluation, implication, aggregation, and defuzzification. In fuzzification, the inputs location, time, role in the system, and frequency of usage are considered crisp numerical values that are converted into a fuzzy membership function. The membership function is the curve that defines the mapping of the membership degree, which ranges between 0 and 1. We used a triangular function given based on three points: $\left(a,0\right),\left(b,1\right),\left(c,0\right)$. Let the membership function defined as follows:

(11) $$\mu a_{i}\left(I_{1}\right)=0\;for\;I_{1}<a_{i}$$

(12) $$\mu a_{i}\left(I_{1}\right)=\frac{\left(I_{1}-a_{i}\right)}{\left(b_{i}-a_{i}\right)}\;for\;a_{i}\leq I_{1}\leq b_{i}$$

(13) $$\mu a_{i}\left(I_{1}\right)=\frac{\left(c_{i}-I_{1}\right)}{\left(c_{i}-b_{i}\right)}\;for\;b_{i}\leq I_{1}\leq c$$

(14) $$\mu a_{i}\left(I_{1}\right)=0\;for\;I_{1}>c$$

where (a,b,c) are the parameters of the membership function, μ is the membership degree, and $a_{i},b_{i},c_{i}$ is the fuzzy set. The fuzzy set is the membership value that ranges between 0 and 1. Here, $a_{i},b_{i},c_{i}$ denote lower bound, mean, and upper bound values. Next, in rule evaluation, the fuzzy operators are used to predict the degree of support for each rule. In this way, the membership function is also generated for a second input. The fuzzy logic is defined as ‘IF antecedent THEN Consequent’. There are three key fuzzy operators as AND $\left(\cap \right),$ which is the intersection for the minimum function; OR (∪), which is for union fuzzy sets representing the maximum function; and NOT (ℸ), which is used to define a complement fuzzy set. It is mathematically denoted as follows:

(15) $$\varphi_{ij}=min\left(\mu_{\alpha i}\left(I_{1}\right),\mu_{\beta j}\left(I_{2}\right)\right)$$

(16) $$\varphi_{ij}=max\left(\mu_{\alpha i}\left(I_{1}\right),\mu_{\beta j}\left(I_{2}\right)\right)$$

(17) $$\varphi_{ij}=1-\left(\mu_{\alpha i}\left(I_{1}\right)\right)$$

In the above, $\varphi_{ij}$ is the firing strength of the rule, and $\mu_{\alpha i}\left(I_{1}\right)$ and $\mu_{\beta j}\left(I_{2}\right)$ are the membership degrees of inputs $I_{1}$ and $I_{2}$ in the fuzzy sets $A_{i}$ and $B_{i},$ respectively. Implication is then performed to refine the output fuzzy set to improve rule strength and is conducted using the rule’s firing strength. Implication works with mamdani, which scales the output membership function based on the rule strength. The rule strength is the value ranging between 0 and 1. I Q-values and ε—greedy are estimated for the selection consequents, with probability, as follows:

$$\varepsilon :a\left(i,j\right)=\mathrm{random}\;\mathrm{action}\;k$$

(18) $$\left(1-\varepsilon \right):a\left(i,j\right)={argmax}_{\left[k\right]}Q\left(i,j,k\right)$$

Here, k is the possible action, i is the index of fuzzy set $I_{1}$ and j is the index of fuzzy set $I_{2}$. Aggregation is then performed by joining all the output fuzzy sets into a single set. The three methods applied are maximum, summation, and probabilistic OR.

(19) $$\mu agg\left(z\right)=max[\mu 1\left(z\right),\;\mu 2\left(z\right),\ldots ,\mu n(z)]$$

(20) $$\mu agg\left(z\right)=\mu 1\left(z\right)+\mu 2\left(z\right)+\cdots +\mu n(z)$$

(21) $$\mu agg\left(z\right)=\mu 1\left(z\right)+\mu 2\left(z\right)-(\mu 1(z)\times \mu 2(z))$$

Implication result and the aggregation of all output is given as,

(22) $$\mu_{ijk\;\left(ij\right)}\left(z\right)=\min\left(\varphi_{ij},\mu c_{k\left(ij\right)}(z)\right)$$

(23) $$\mu agg\left(z\right)=\max\left\{\mu_{11_{k\left(11\right)}}\left(u\right),\;\mu_{12_{k\left(12\right)}}\left(u\right),\ldots ,\mu_{{ij}_{k\left(ij\right)}}\left(u\right),\ldots \right\}$$

where u is the output variable, and $\mu_{{ij}_{k}}\left(u\right)$ is the firing strength to the output fuzzy set for action k. Defuzzification is the final step, which converts the fuzzy set into crisp values for decision-making regarding attribute count. In this work, only two choices of outcome are considered: either all attributes or half of the attribute set. The defuzzifier operates in using weighted average. The u* is the crisp output, formulated as

(24) $$u*=\frac{\left(\sum ij\varphi_{ij}\cdot u_{k(ij)}\right)}{\left(\sum ij\varphi_{ij}\right)}$$

Finally, the Q-values are updated using the following equation:

(25) $$Q\left(i,j,k\left(i,j\right)\right)=Q\left(i,j,k\left(i,j\right)\right)+\alpha \cdot \varphi_{ij}\cdot [r+\gamma \cdot {max}_{\left(k\right)}Q^{\prime }\left(i,j,k\right)-Q\left(i,j,k\left(i,j\right)\right)]$$

where k(i,j) is the action that is selected for rule $R_{ij}$, α is the learning rate, γ is the discount factor, r is the reward, and $Q^{\prime }\left(i,j,k\right)$ represents the Q-values in the next state. From the selection of attributes for the received set of requests, parallel matching for attribute access control is performed using the GNN.

Let us consider two graphs $G_{1}=\left(V_{1},E_{1}\right)$ and $G_{2}=\left(V_{2},E_{2}\right)$ with attribute features, and let $X_{1}$ and $X_{2}$ be the node feature matrices. The matching function is defined as

(26) $$f0:V_{1}\times V_{2}\to [0,1]$$

This describes the attributes and their corresponding probability. Let the user node and resource node be represented as $U=\{u_{1},u_{2},\ldots \ldots ,u_{m}\}$, $R=\{r_{1},r_{2},\ldots \ldots ,r_{n}\}$; then, the node set is V=U∪R.

Attribute embedding is presented for each node, comprising an attribute vector with dimension d. The user attributes and resource attributes are $X_{U}\in R^{m\times d}$ and $X_{R}\in R^{n\times d}$, respectively. The final combined attribute matrix is

(27) $$X\in R^{(m+n)\times d}$$

The edge of the graph denotes the relationship between users and the resource and is constructed as follows:

(28) $$E=\{(u_{i},r_{j})|u_{i}\to r_{j}\}$$

where $u_{i}\to r_{j}$ indicates that user i has attempted to access the resource  j. The GNN architecture is composed of three layers: a graph convolution layer, graph attention layer, and output layer. The operation carried out in the graph layer is given mathematically as follows:

(29) $$H^{\left(1\right)}=\sigma ({\tilde{D}}^{-\frac{1}{2}}{\tilde{A}\tilde{D}}^{-\frac{1}{2}}{XW}^{\left(0\right)})$$

Here, the adjacency matrix is $A\in {\left\{0,1\right\}}^{\left(m+n\right)\times \left(m+n\right)},$ which shows the connections between nodes; $\tilde{A}=A+I$, I is the self-loops; $\tilde{D}$ is the degree matrix of $\tilde{A}$; the weighted matrix is $W^{\left(0\right)}\in R^{d\times h}$; σ is the non-linear activation function as in recurrent layer; and $H^{\left(1\right)}\in R^{(m+n)\times h}$ is the hidden layer node. The attribute information of users and resources is exchanged in this layer, which aggregates information with its neighboring nodes.

In the graph attention layer, the weighted node relationship is given as

(30) $$H^{\left(2\right)}=GAT(H^{\left(1\right)},A)$$

(31) $$\alpha_{ij}=\frac{exp\left(LeakyReLU\left(\alpha^{T}[W^{\left(1\right)}h_{i}^{\left(1\right)}||W^{\left(1\right)}h_{j}^{\left(1\right)}]\right)\right)}{\sum_{k\in N_{i}}exp\;\left(LeakyReLU\left(\alpha^{T}[W^{\left(1\right)}h_{i}^{\left(1\right)}||W^{\left(1\right)}h_{k}^{\left(1\right)}]\right)\right)}$$

(32) $$h_{i}^{\left(2\right)}=\sigma \left(\sum_{j\epsilon N_{i}}\alpha_{ij}W^{\left(1\right)}h_{j}^{\left(1\right)}\right)$$

where $W^{\left(1\right)}$ is the weighted matrix, α is the attention vector, || is concatenation, and $N_{i}$ represents the neighborhood node i. From this layer, the most relevant attributes and their relationships are determined for making access decisions. Then, the output layer takes final decision, which is based on mapping as follows:

(33) $$Z=softmax(W^{\left(2\right)}H^{\left(2\right)}+b^{\left(2\right)})$$

where the probability to allow or deny access is $Z\in R^{\left(m+n\right)\times 2},$ and $W^{\left(2\right)}$ and $b^{\left(2\right)}$ are the weighted matrix and bias, respectively. The training process is defined based on the loss function as follows:

(34) $$L=-\frac{1}{\left|Y\right|}\sum_{i\in Y}\sum_{c=0}^{1}y_{i,c}\log\left(z_{i,c}\right)$$

The Adam optimizer is used to minimize the loss:

(35) $$\theta_{t+1}=\theta_{t}-\eta \cdot \frac{{\hat{m}}_{t}}{\sqrt{{\hat{v}}_{t}}+\epsilon }$$

The parameters for the loss function are θ and η, representing the model parameters and learning rate, respectively; while ${\hat{m}}_{t}$ and ${\hat{v}}_{t}$ are bias-corrected estimates of the first and second moments, respectively; Y is the set of nodes; and $y_{i,c}$ and $z_{i,c}$ are the true label and predicted probability, respectively. $y_{i,c}$ is either 1, indicating that access is allowed, or 0, meaning access is denied. Following the training of the GNN, the node attributes are processed, and the output probability is predicted (Algorithm 2).
**Algorithm 2.** Final Access Decision Rule1. Begin2. If argmax $\left(Z_{r_{j}}=1\right)$         {        It allows access      Else if argmax $\left(Z_{r_{j}}=0\right)$       {           It denies access       }    End if         }3. Stop

**Figure 6:**
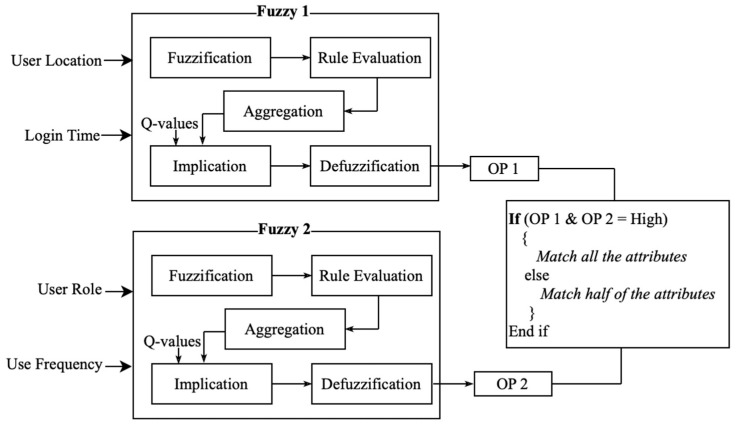
Bi-fuzzy Q-learning model.

To process the input data in parallel, fuzzy logic is introduced in the form of Bi-Fuzzy logic, where the set of inputs are processed in parallel to minimize the processing time as depicted in Figure 6. This Bi-Fuzzy approach is integrated with Q-learning to update the rules according to the environment. This combination can handle dynamic environments with limited tuning requirements, since Q-learning uses a learning rate that responds to small variations in the input state while fuzzy logic preserves the decision structure. The GNN is, therefore, the best choice for scalability, as it can perform parallel computations while detecting multiple attributes. According to the chosen number of attributes, the received number of user requests attributed are validated, and access is provided or denied.

The interpretability of the proposed mechanism is primarily achieved through the Bi-Fuzzy–Q-learning component. Fuzzy membership functions and linguistic rules (e.g., low, medium, high trust/risk) are used to dynamically select authentication attributes, enabling transparent and explainable decision logic. The GNN is employed only as a feature extractor, while the final decision is governed by fuzzy rules, which enhances interpretability within the simulated environment. Attributes are selected to minimize the verification time while still supporting the access decision according to the request context. Based on the request, the specific device is allowed or denied access to the data based on the attributes selected for validation. Therefore, the reduced validation level is not intended to bypass essential security conditions but to offer a simplified dynamic policy within the scope of the present study.

### 4.4. Hyperledger Fabric Blockchain

In the proposed system, Hyperledger Fabric (HLF) blockchain is used to store the security credentials and is extracted whenever validation is essential. In the proposed HADA framework, the HLF blockchain stores only hashed authentication credentials (e.g., identity, certificates, challenge–response values) and access-related credential hashes, which are validated by distributed peer nodes in a permissioned ledger to ensure integrity, immutability, and decentralized trust. The credentials are converted into hashes using the lightweight SPONGENT algorithm.

The reason for choosing the HLF blockchain is that it supports both confidential transactions and scalability. It can handle thousands of transactions per second, so it can process many requests coming into the system. To minimize computation during hashing, a lightweight hashing algorithm that increases security is preferred.

This algorithm starts with padding and initialization, followed by an absorption phase and internal permutation function (S-Box layer, Bit permutation, and linear mixing). The padding and initialization step is formulated below.

(36) $$c_{x}^{*}=pad\left(c_{x}\right)$$

(37) $$\mathrm{Internal}\;\mathrm{State}:\;S_{0}=V$$

where $c_{x}$ is the input credential that is padded using the standard padding rule as pad 10*1. This padding ensures that the length becomes a multiple of the bit rate r, while V represents a fixed initialization vector. The padded message, which splits into t blocks at size r, is mathematically represented as follows:

(38) $$c_{x}^{*}=m_{0}\left|\left|m_{1}\right|\right|\ldots ||m_{t-1}$$

Let S and c be the state and the security credential that is to be hashed. The input $c_{x}$ is padded to match the specified block size. $S_{0}$ is intitalized as the state with the vector V, and c is split into $c_{x}$ blocks. Next, each block is absorbed into the state S using the bitwise XOR operator:

(39) $$S_{i+1}=\;S_{i}⊕c_{x}$$

Then, the S-box and bit permutation layers are applied, in which non-linearity is introduced while using the S-box. The process of bit permutation influences the overall state. A generalized Feistel Structure is applied for each round based on the three following steps.

(i) S-Box Layer:
  (40) $$S^{\prime }=S⊕SBox(S)$$
(ii) Bit Permutation:
  (41) $$S^{\prime }=P(S)$$
(iiI) Mixing Layer: To enhance the cryptanalysis, an additional mixing function is performed that enables updates to the changes throughout all states.

Finally, in the squeezing phase, the credential is converted into hashes after all rounds are complete. The hash value is given as

(42) H=trunc (S,h)

These hash values are stored in the HLF blockchain, which is maintained for each DU and DO in the block structure. The SPONGET algorithm is measured according to preimage resistance, second preimage resistance, and collision resistance, which are given as $O{\left(2^{C}\right)}^{n}$, $O{\left(2^{C}\right)}^{n}$ and $O{\left(2^{n/2}\right)}^{n},$ respectively, where C is the capacity and n is the output length.

## 5. Experimental Analysis

In this section, the development and evaluation of the proposed system are described.

### 5.1. Simulation Setup

The proposed HADA-IoT control mechanism was developed in iFogSim [39] using Java Development Kit, Wamp server, and NetBeans. We note that the HLF block generation behavior was not simulated natively within iFogSim. Instead, it was represented through a custom discrete-event extension integrated into the iFogSim simulation loop to reflect the blockchain-related workflow within the implemented HADA framework. We developed the system model to include the data owner, data user, certificate authority, and HLF blockchain. The simulation specifications used in this work are provided in Table 3.

It should be noted that the simulation environment, with two data owners and six data users, was used for controlled validation of the proposed authentication and access control mechanism. Therefore, the performance results should be interpreted as preliminary efficiency results rather than as full scalability evidence for large-scale IoT deployment. Since the work is simulation-based, reproducibility depends on the reported implementation settings, parameters, and workflow assumptions used in this study.

**Figure 7:**
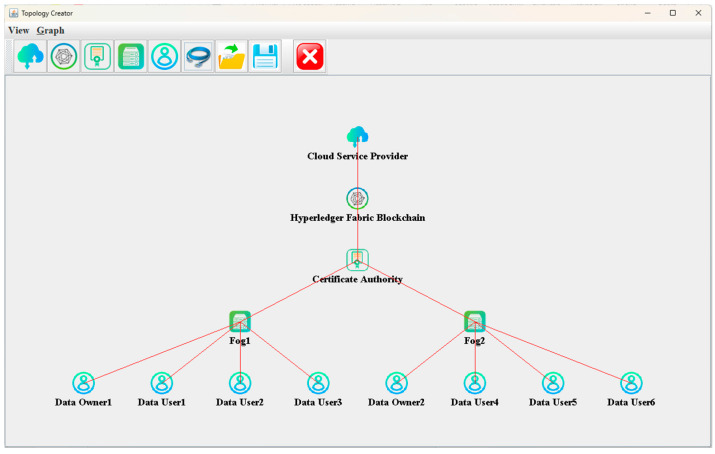
Designed HADA system in iFogSim.

According to the specifications, the HADA-IoT system design is illustrated in Figure 7. This architecture is applied with the processes of registration, authentication, and control. Upon successful authentication, the sample data are uploaded for testing and accessed. Lightweight hashing is incorporated in the HLF blockchain technology.

### 5.2. Comparative Analysis

The comparative analysis described in this section is an evaluation of the proposed method against previous research works. The main parameters measured are throughput, latency, resource utilization, and accuracy. The mathematical definitions of these metrics are provided. In this work, these metrics were measured in the simulation environment during the execution of registration, authentication, credential validation, and attribute-based access control within the proposed HADA workflow.

#### 5.2.1. Performance of Throughput

Throughput is defined as the number of requests that are successfully processed over a specific time period. The proposed HADA system model measures the number of successful authentication requests that are executed in the given time. The mathematical formulation for throughput measurement is given as

(43) $$Th=\frac{{SA}_{r}}{t}$$

**Figure 8:**
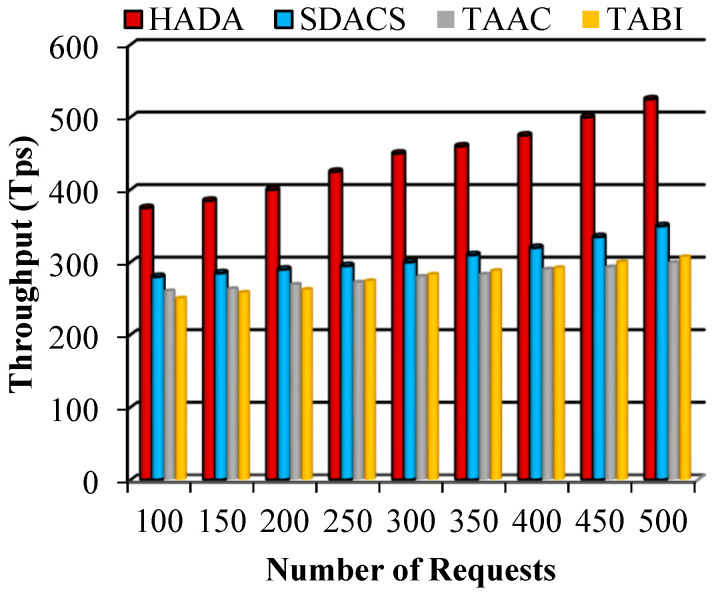
Graphical plot of throughput.

In Figure 8, the throughput performance of the proposed HADA system is compared with multiple existing schemes. Higher throughput indicates more efficient processing of authentication requests. The results show that the HADA system achieves consistently higher throughput than the compared methods, including the SDACS scheme. In particular, when compared with SDACS, the average throughput increases from approximately 307 TPS to 443.89 TPS, representing an improvement of nearly 100 TPS. This improvement reflects the efficiency of the proposed lightweight authentication and access control mechanisms in handling authentication requests.

#### 5.2.2. Latency Performance 

Latency is a significant measure that is calculated based on the time delay. It is defined as the time delay between sending the request for attribute access and receiving the response indicating successful attribute access. The mathematical formulation for the estimation of latency is given as

(44) $$L=RES\left(t\right)-REQ(t)$$

Here, latency L is determined from $RES\left(t\right)$ and $REQ\left(t\right),$ which are the response time and request time, respectively. The decreased latency achieved with the proposed system supports a scalable system. The proposed HADA system is intended to receive multiple requests for authentication and attribute access control. The lightweight algorithm employed minimizes computation and predicts the number of attributes for matching. 

**Figure 9:**
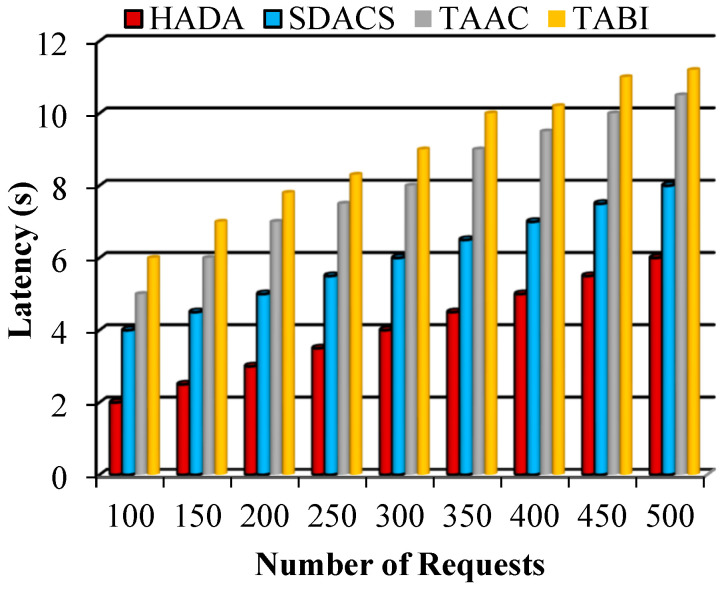
Graphical plot of latency.

In Figure 9, the latency performance of the proposed HADA system is compared with multiple existing schemes. Lower latency indicates reduced processing time for authentication and attribute access control requests. The results show that the HADA system consistently achieves lower latency than the compared methods, including the SDACS scheme. This improvement reflects the reduced computational overhead of the proposed lightweight authentication and dynamic attribute selection mechanisms.

#### 5.2.3. Performance of Resource Utilization

Resource utilization is the key to predicting the ability of the system to process requests from data owners and users. The resources CPU and memory were considered in the comparison and are calculated using the following equations:

(45) $$CU=\left(\frac{CPU\;(t)}{T(t)}\right)\times 100$$

where $T\left(t\right)=CPU\left(t\right)+I(t)$

(46) $$MU=\left(\frac{Mu}{TM}\right)\times 100$$

Consider CU as the CPU utilization, computed using CPU (t), $T\left(t\right),$ and I$\left(t\right)$—the CPU time, total time and idle time. MU is the memory utilization, computed using Mu and TM, representing the memory used and total memory. The memory used is $\left(TM-Free\;Memory\right)$.

**Figure 10:**
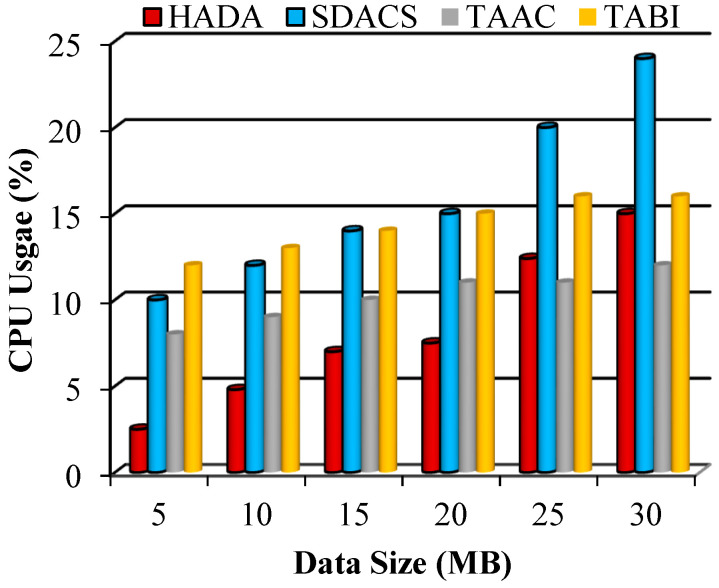
Graphical plot of CPU utilization.

**Figure 11:**
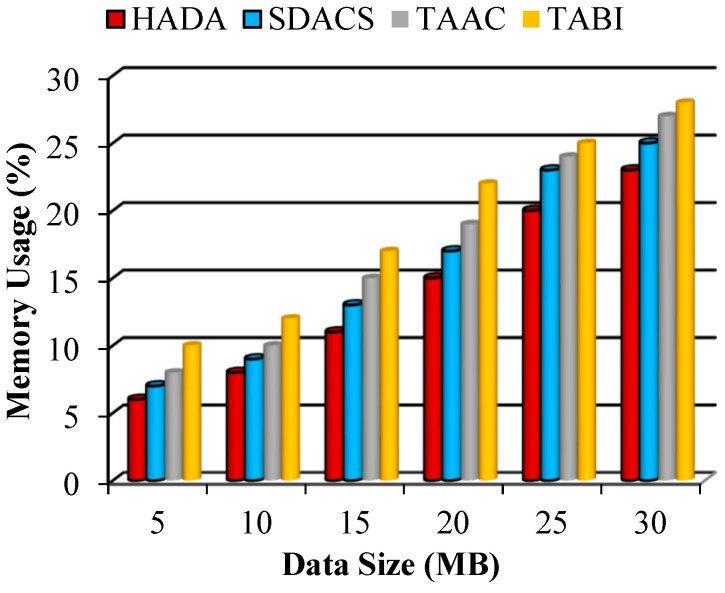
Graphical plot of memory utilization.

The systems’ performance with respect to CPU and memory utilization is illustrated shown in Figure 10 and Figure 11, respectively. In this work, fog nodes are appointed as edge devices that distribute the requests to the certificate authority. The certificate authority requires memory, CPU, and storage to operate. We considered the amount of memory and CPU resources required to process the volume of user data that arrives. Based on the comparison, the proposed system uses fewer resources than existing systems, indicating that the proposed method provides a processing advantage.

An average estimation of CPU resource utilization is about 8.2% from the fully available CPU capacity whereas it is approximately 15.83% of CPU used, so the proposed is 5% to 6% lesser in the resource utilization. The selected number of attributes process using parallel processing algorithm of GNN optimizes the CPU utilization.

The memory utilization achieved by the proposed HADA system is 13.83%, while it is 15.67% in the SDACS scheme. Overall, the proposed approach demonstrates lower memory consumption compared to the evaluated schemes. The observed reduction of approximately 2% in memory utilization compared to SDACS indicates improved efficiency, which supports the suitability of the proposed system for handling large data sizes in resource-constrained IoT environments.

#### 5.2.4. Performance of Accuracy

**Figure 12:**
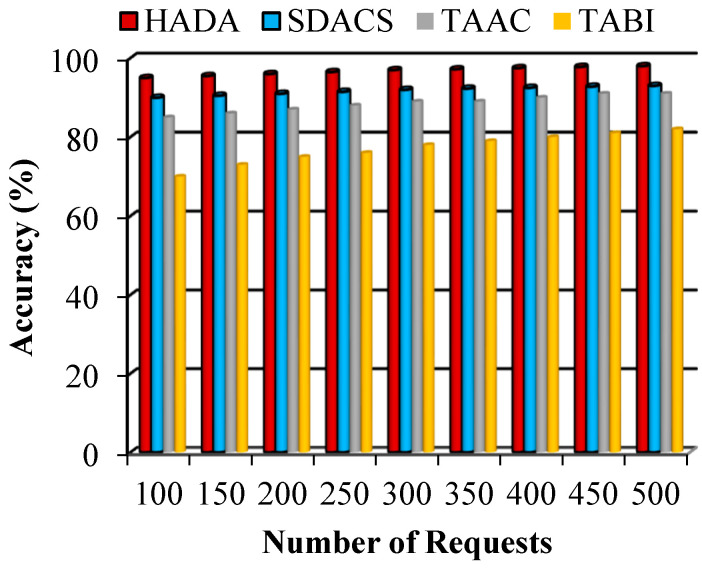
Comparison of accuracy.

Accuracy is one of the common measurement parameter that enables to determine exactness of authentication. It is able to differentiate legitimate devices and illegitimate devices. In this proposed HADA system the accuracy value defines the successful authentication of the device requests that includes both data owners and users. The correctness of the authentication scheme is demonstrated from this accuracy. Figure 12 shows comparison of accuracy of HADA system and the evaluated schemes including SDACS. In this comparison, HADA system achieves higher accuracy compared to the evaluated schemes due to the incorporation of simpler authentication using lightweight hashing algorithm in blockchain.

The current evaluation focuses on comparative behavior against related access control schemes within the implemented simulation environment. Platform-level blockchain benchmarking using tools such as Hyperledger Caliper, as well as ablation-based comparison by removing individual modules of the proposed framework, are not included in the present study and are considered as future extension directions.

### 5.3. Complexity Analysis

The complexity is determined for SPONGENT and SKINNY algorithm which are used for hashing and data encryption, respectively. Both are lightweight methods that enable the incorporation of limited computation and resources for processing.

#### 5.3.1. Analysis of SPONGENT Algorithm

Step 1: AddConstants—with O (1) that performs XORs to add round constants to the state.

Step 2: Substitution Layer—4-bit S-boxes are applied to the state The time taken here is O (b/4) operations (one S-box per 4 bits).

Step 3: Permutation Layer—bits are permuted according to a fixed pattern, and computation comprises O(b) operations (one operation per bit).

Then, permutation complexity is O(R × b), the absorption phase is O(⌈m/r⌉ × R × b) and the squeezing phase is O(⌈n/r⌉ × R × b); therefore, the total time complexity of the SPONGENT algorithm is O((⌈m/r⌉ + ⌈n/r⌉) × R × b).

#### 5.3.2. Analysis of SKINNY Algorithm

Step 1: SubCells—S-boxes are applied to each byte of the state, and O(n/8) operations (one S-box lookup per byte) is the time complexity.

Step 2: AddConstants—XORs round constants to the state with O(1) operations (constant time regardless of block size).

Step 3: AddRoundTweakey—XORs the round tweakey to the state with O(n/2) operations (tweakey is added to half of the state).

Step 4: ShiftRows—bytes are permuted in the state, and O(n/8) operations (one shift per byte) are executed.

Step 5: MixColumns—binary matrix multiplication with O(n) operations (each column requires a fixed number of XOR operations).

The round complexity is O(n), and the total complexity is given as O(r × n). For SKINNY-64-64: T = O(32 × 64) = O(2048) = O(n^2^).

These theoretical complexity characteristics are reflected in the experimental results presented in Section 5.2, where reduced latency and lower CPU and memory utilization are observed, confirming the suitability of the proposed lightweight algorithms for resource-constrained IoT devices.

### 5.4. Security Analysis

The following security discussion focuses on the main security properties supported by the proposed HADA framework, namely confidentiality, integrity, trustworthiness, non-repudiation, and authorization. Within this analysis, the related attack resistance is discussed in connection with these security properties.

#### 5.4.1. Confidentiality

In the proposed HADA system, the security constraint of confidentiality is assured since the system authenticates the data owner and user. A hybrid authentication method is proposed in which unique credentials are used for authentication. Then, only authorized owners and users are allowed into the system to access data. This proposed system ensures that data remain confidential, preventing replay and eavesdropping attacks.

#### 5.4.2. Integrity

Integrity is the key constraint in security analysis and is achieved when the data are unaltered. This aspect of security is ensured in the HADA system, which encrypts data using lightweight SKINNY algorithm. The proposed lightweight algorithm enables safer storage of data with minimal computation. Integrity is maintained by combining timestamp–nonce validation with session-specific parameters, which collectively prevent replay attacks, timestamp synchronization exploitation, message tampering, and man-in-the-middle modification attempts.

#### 5.4.3. Trustworthiness

Trustworthiness defines the behavior of the sensor node as honest and reliable. The existence of malicious and compromised nodes in the network breaks trustworthiness. In the proposed HADA system model, the authentication credentials include trust estimation of each node, which ensures the trustworthiness of the system. This trust value indicates that the sensor node is not malicious. Assured trustworthiness enables the system to overcome blackhole, gray hole, sink hole, and spoofing attacks.

#### 5.4.4. Non-Repudiation

Non-repudiation is a security property that ensures that a sensor node cannot deny transmitting and receiving. In the HADA system, hybrid authentication allows only registered users and restricts malicious nodes because it considers the trust value, which is computed based on the behavior of the sensor node.

#### 5.4.5. Authorization

Authorization occurs when only legitimate sensor nodes are permitted access. In the HADA system, the sensor nodes are allowed to access the data only after the attributes are satisfied. The hybrid authentication and attribute access control mechanism provides authorization for HADA system model.

## 6. Conclusions

In this paper, a novel hybrid authentication and dynamic attribute access control scheme is presented for IoT environments. Both the data owner and user must be authenticated for each data upload and access. This authentication is carried out with multiple factors that are securely exchanged using differential privacy, and trust is estimated. The lightweight SKINNY algorithm is used for data encryption, and SPONGENT is used for hashing credentials in the HLF blockchain. Then, to access data from the cloud, the system employs a dynamic attribute access control mechanism that selects the number of attributes dynamically using a Bi-Fuzzy Q-learning algorithm. Then, the graph neural network is applied to match the attributes and make the decision to allow or deny access. We incorporated a fabric ledger blockchain for scalable and latency, as it minimizes the processing of the received requests. Despite these advantages, this study has certain limitations. Experimental validation was conducted in a small simulation environment, platform-level benchmarking of the Hyperledger layer was not included, and a formal security proof was not carried out. These limitations will be addressed in the next stage of this work through larger-scale validation, stronger theoretical analysis, and further optimization for practical real-time IoT deployment. Additionally, in future work, formal security validation using game-based analysis or protocol verification tools will be considered to further strengthen the theoretical security assurance of the proposed HADA framework. We also plan to present a dynamic attribute policy that enables updates to new policies based on learning previous decisions of the attribute access control method and to further optimize the framework for practical real-time IoT deployment.

## Figures and Tables

**Table 1:** Challenge–response pair.

Challenge	Response
$C_{1}$	$R_{1}$
$C_{2}$	$R_{2}$
$C_{3}$	$R_{3}$
⋮	⋮
$C_{k}$	$R_{k}$

**Table 2:** Challenge–response status prediction.

Challenge	Response	Status
Valid Challenge	Valid Response	Success
Invalid Challenge	Error Response	Failure
Repeated Challenge	Consistent Response	Verified
Modified Challenge	New Response	Valid
Corrupted Challenge	No Response	Error

**Table 3:** Simulation specifications.

**Model Parameters**	**Count**
Number of DO	2
Number of DU	6
Number of CA	1
Cloud Service Provider	1
**Hardware and Software**	**Version**
Java Development Kit	23.0.2
Netbeans IDE	24
Wamp Server	3.2.6
MySQL	5.7.36
RAM	16.0
Processor	11th Gen Inter (R) Core (TM) i5
System Type	64-bit
**HLF Blockchain Specs**	
Block size	1–2 MB
Block generation time	0.5–2 s
Number of channels	1 or more
Number of peer nodes	2 to 4
Endorsement Execution time	5–20 ms

## Data Availability

No new data were created or analyzed in this study.
